# Investigation of K14/K5 as a Stem Cell Marker in the Limbal Region of the Bovine Cornea

**DOI:** 10.1371/journal.pone.0013192

**Published:** 2010-10-06

**Authors:** Bo Chen, Shengli Mi, Bernice Wright, Che John Connon

**Affiliations:** School of Pharmacy, University of Reading, Reading, United Kingdom; Johns Hopkins University, United States of America

## Abstract

**Background:**

Identification of stem cells from a corneal epithelial cell population by specific molecular markers has been investigated previously. Expressions of P63, ABCG2 and K14/K5 have all been linked to mammalian corneal epithelial stem cells. Here we report on the limitations of K14/K5 as a limbal stem cell marker.

**Methodology/Principal Findings:**

K14/K5 expression was measured by immunohistochemistry, Western blotting and Real time PCR and compared between bovine epithelial cells in the limbus and central cornea. A functional study was also included to investigate changes in K5/14 expression within cultured limbal epithelial cells undergoing forced differentiation. K14 expression (or its partner K5) was detected in quiescent epithelial cells from both the limbal area and central cornea. K14 was localized predominantly to basal epithelial cells in the limbus and suprabasal epithelial cells in the central cornea. Western blotting revealed K14 expression in both limbus and central cornea (higher levels in the limbus). Similarly, quantitative real time PCR found K5, partner to K14, to be expressed in both the central cornea and limbus. Following forced differentiation in culture the limbal epithelial cells revealed an increase in K5/14 gene/protein expression levels in concert with a predictable rise in a known differentiation marker.

**Conclusions/Significance:**

K14 and its partner K5 are limited not only to the limbus but also to the central bovine cornea epithelial cells suggesting K14/K5 is not limbal specific *in situ*. Furthermore K14/K5 expression levels were not lowered (in fact they increased) within a limbal epithelial cell culture undergoing forced differentiation suggesting K14/K5 is an unreliable maker for undifferentiated cells *ex vivo*.

## Introduction

The cornea is the transparent tissue at the front of the eye; corneal epithelial cells, which are its external cell layers, are supported by corneal epithelial stem cells, located in the limbus, an area where the central cornea and conjunctiva meets. Corneal epithelial stem cells give rise to renewal of the corneal surface, as part of normal cell death or repair [1], [2], [3], [4]. The mechanisms that control corneal epithelial stem cell differentiation are not fully understood, although several cell signaling pathways have been proposed to regulate corneal epithelial stem cell activities, as others have shown [5], [6], [7]. Identification of the epithelial stem cells from a corneal cell population by specific molecular markers has been intensively investigated for over the last 10 years. P63 [8], ABCG2 [9] and K14 [10] have been reported to be positively expressed in mammalian corneal epithelial stem cells, and K3 [11], [12], K12 [13], and Cx43 [14] have been shown to be only positively expressed in terminally differentiated corneal epithelial cells. However, there still remains no decisive stem cell marker. Therefore, to identify corneal epithelial stem cells a combination of various cell makers including both positive and negative putative corneal epithelial stem cell makers must be used [15], [16], [17], [18].

P63, ABCG2 and K14 have been shown to accumulate in basal cells within the limbus. Histological [19] and molecular [1] studies of limbal basal cells has demonstrated that these cells have typical adult somatic stem cell features, such as small cell size, large nuclear to cytoplasm ratio [3], and ability to form holoclones [20]. Limbal epithelial basal layers are also responsible for corneal epithelial wound healing in mammalian cornea [1], [2], [3], [4], further proof that, corneal limbal stem cells are believed to be exclusive to the limbus. These stem cells can provide daughter cells, which populate the corneal epithelium centripetally, differentiating as the move from the limbal area of the limbus to central cornea. However, recently it has been shown that after serial transplantation, mouse corneal epithelium is self-maintained suggested from that central cornea epithelium contains oligopotent stem cells [4]. This result conflicts with the previous limbal basal epithelial stem cell hypothesis, and subsequently questions the reliability of several limbal specific corneal epithelial stem cell markers.

Keratin 14 has been proposed as a marker for epidermal stem cells in skin [21], [22], and it has also been shown that its expression highlights the basal cells of limbal epithelium with its keratin pair partner, K5. K5/14 pairs have been shown positive to bovine limbal basal layers, suggesting K5/14 as a potential corneal stem cell marker [23], [24], [25].

However, in the rabbit cornea, K14 positive cells were confined not only to the basal but also to the suprabasal layers of the limbal epithelium [26]. This discrepancy highlights the difficulty of interpreting data obtained from K5/K14 in human and animal models.

In this paper, our hypothesis is that a traditional corneal epithelial stem cell marker, K14 is not ideal for the identification corneal epithelial stem cell especially in expanded cells. We report here that K14 expression was observed in both bovine limbus and central cornea and that K14 expression was found to increase in limbal epithelial cells, after air-lifting treatment, a cell culture technique well known to increase cell differentiation and promote cell-cell barrier function [18], [27], [28], [29].

## Materials and Methods

### Preparation of human AM as substrate for limbal stem cells expansion

Cryopreserved human AM was collected from the Division of Ophthalmology and Visual Science, Queens Medical Centre, Nottingham, UK with approval from the Local Research Ethics Committee (Nottingham). The membranes were washed with sterile phosphate-buffered saline (PBS) containing penicillin (100IUnits/mL) and streptomycin (100 µg/mL) (Gibco; UK) within a class II microbiology safety cabinet, before they were cut into pieces (approximately 4 cm^2^) and immediately stored at −80°C in PBS. Before use, the AM was thawed and washed in sterile PBS. AM was positioned with its native epithelium side up within transwell inserts held inside six well culture plates (Corning; UK).

### Bovine corneal epithelial cells preparations

Bovine cornea has a structure and biochemical makeup similar to human cornea. Like human cornea bovine corneal stem cells have been localized to the basal layers of the limbus [10], and have successfully been used as an alternative source of human limbal stem cells for basic research [18], [30]–[32]. Bovine eyes were collected (Chity wholesale abattoir, Guildford, UK) within 2 hours of death and transported on ice before corneas were dissected for epithelial cell isolation. The whole limbal ring tissue from each eye, including the subconjunctival Tenon's capsule, was cut into 10–12 pieces (approximately 5 mm long) and incubated for 12 hours at 37°C in basal culture medium with 0.02% type IA collagenase (Sigma-Aldrich; UK).

The epithelium was peeled from the enzyme-treated limbal pieces, and incubated with 0.05% trypsin/EDTA for 10 min at 37°C, and the cell sheets were dissociated into single cells by agitation through a 22 gauge needle. The suspension of corneal limbal epithelial cells, was placed in to supplemented media (Dulbecco's modified Eagle's medium and Ham's F12 (1∶1) (DMEM/F12) (Gibco; UK) including 5% fetal bovine serum (FBS), 2 ng/mL human epidermal growth factor (hEGF), 5 µg/mL insulin, B27 supplement [31] medium and penicillin streptomycin (Invitrogen; UK), and seeded onto either AM (spread on the bottom of culture inserts). AM and cells were submerged in supplemented media for 2 weeks and then exposed to air by lowering the medium level (air-lifting) for 1 week to promote corneal epithelial cells differentiation. Cultures were incubated at 37°C under 5% CO_2_ for approximately 3–4 weeks; media was replaced every 2 days.

### Immunohistochemistry

Fresh bovine corneal pieces (dissected from bovine eye balls) and limbal epithelial cells expanded upon AM (collected using a 6 mm biopsy (Brymill, UK) before (2 weeks) and after air-lifting (3 weeks), were embedded in tissue Tek O.C.T. compound (Agar Aids; UK), snap-frozen in liquid nitrogen and stored at -80°C prior to being sectioned. Cryostat sections (7 µm thick) were placed onto poly-lysine coated slides and air-dried for 2 hours. Slides were then fixed in 100% methanol at −20°C for 15 min and in acetone (−20°C) for 5 min before they were washed in PBS. Tissue sections were incubated with 1% goat serum or bovine serum albumin (BSA) at room temperature for 30 min to block non-specific binding.

Blocked sections were incubated in anti-K14 (1∶100,guinea Pig, polyclonal, Progen, Germany), anti-K3 (1∶50, mouse,monoclonal, Chemicon, UK), anti-integrin α6 (1∶100, mouse, monoclonal, Progen, Germany) primary antibodies overnight at 4°C and washed for 5 min in PBS. Horse anti-mouse (1∶50, Vector Labs, UK)/Goat anti-guinea pig (1∶100, Progen, Germany) secondary antibodies conjugated to fluorophores were applied for 60 minutes at room temperature. Unbound secondary antibodies were removed by washing in PBS and sections were mounted under glass cover slips using DAPI mounting media (Vector Labs, U.K.). Negative controls were performed by replacing the primary antibody with PBS. Mounted sections were examined by fluorescence microscopy (Imager A1, Zeiss, Germany).

### Isolation of RNA and cDNA synthesis

Total RNA was isolated from cells cultured on AM (n = 6), from fresh bovine cornea (central and limbal region, n = 4) using the TRI reagent (Sigma, Poole, UK), according to the manufacturer's protocol. Total RNA was quantified spectrophotometrically (GE Healthcare, UK), and 1 ng RNA was reverse-transcribed using RevertAid H Minus First Strand cDNA synthesis Kit (Fermentas, U.K.), following the manufacturer's protocol.

### Real-time quantitative PCR (qPCR)

A custom-made PerfectProbe assay (PrimerDesign, U.K.) was used to quantify Keratin 12 gene (accession number: XM_001255461) and Keratin 5 (accession number: AF102774) expression. Each reaction was performed in triplicate on three different replicate samples with a final reaction volume of 20 µL containing 10 µL 2X qPCR Mastermix (Primerdesign, U.K.), 1 µL reconstituted perfectprobe primer/probe mix (Primerdesign, U.K.), 4 µL PCR-Grade water (Primerdesign, U.K.) and 5 µL cDNA (1∶10 of original concentration). Non-template controls were also run. Real-time reactions were performed within a 96-well plate (Fisher, U.K.) in the ABI PRISM 7700 Sequence Detector (Applied Biosystem, U.K.). The real time PCR data was analyzed by delta-delta CT-method; therefore, the K12 and K5 mRNA expression level were approximate.

### Western blotting

Proteins from bovine limbal cells, central corneal epithelial cells, and epithelial cells grown on AM with and without air-lifting (4 µg total protein for each condition; estimated using the modified Lowry assay), were separated by one-dimensional sodium dodecyl sulphate-polyacrylamide gel electrophoresis (SDS-PAGE) using 10% gels. They were transferred to polyvinylidine difluoride (PVDF) membranes and non-specific binding to membranes was blocked by incubation with 5% (w/v) milk dissolved in 1X Tris-buffered saline-Tween (TBS-T) (20 mM Tris-base, 0.14 M NaCl, 0.1% Tween®-20; pH 7.6). Membranes were incubated with mouse anti-K3, anti-K14 and anti-GAPDH primary antibody (1 µgmL^−1^) diluted in 2% (w/v) milk dissolved in 1X TBS-T at 4°C overnight. Blots were washed for 45 min in 1X TBS-T before incubation with an anti-mouse HRP-conjugated secondary antibody (1∶6000 dilutions) for 2 h at room temperature. Proteins were detected on X-ray film using an enhanced chemiluminescence (ECL) system.

### Statistical analysis

Student's t-tests (unpaired) were performed using Microsoft Excel. Results are presented as the mean of 3 individual experiments with standard error of mean (S.E.M.) and *P*-value ≤0.05 was considered significant.

## Results

### Location of Keratin 14 and Keratin 3 expression *in vivo* bovine cornea

**Figure 1:**
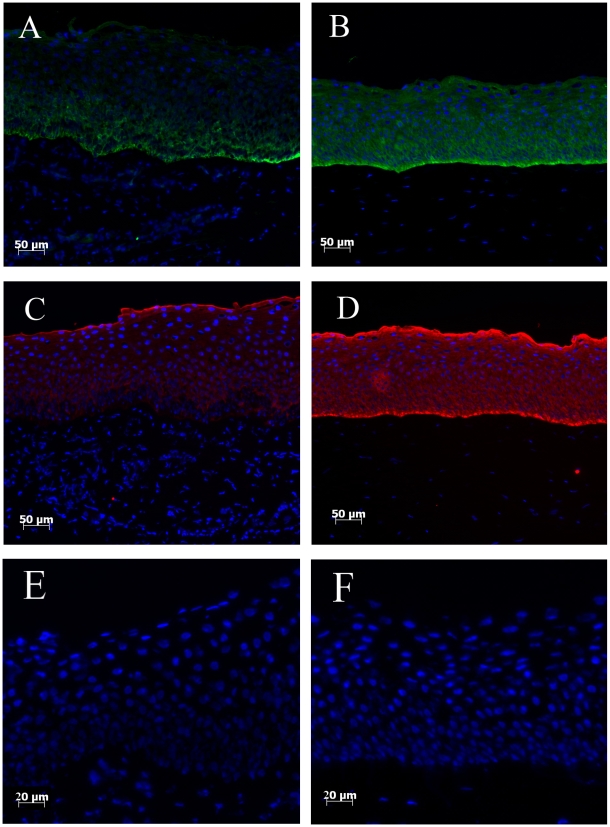
K14 and K3 expression in limbal and central corneal regions. K14 (Green) positive cells were only detected to the basal layer of limbus (A), but its expression was also seen throughout central corneal layers (B). Although K3 (Red) expression is absent in the limbal basal layer cells (C), K3 highlights whole central corneal cell layers (D). Control sections for limbal (E) and central cornea (F) show no background staining.

K14 positive cells were observed in the basal layers of limbus, whereas K14 expression was not detected in the superior cell layers (Figure 1A). In contrast, within the central cornea, K14 positive cells were distributed throughout the epithelial cell layers (Figure 1B). Epithelial cells stained with K3 were detected in limbal superior layers, but absent in the basal cell layers (Figure 1C); K3 positive cells were highly expressed in the central cornea, and present throughout all the epithelial cell layers (Figure 1D).

### Quantification of Keratin 14 and Keratin 3 expression

**Figure 2:**
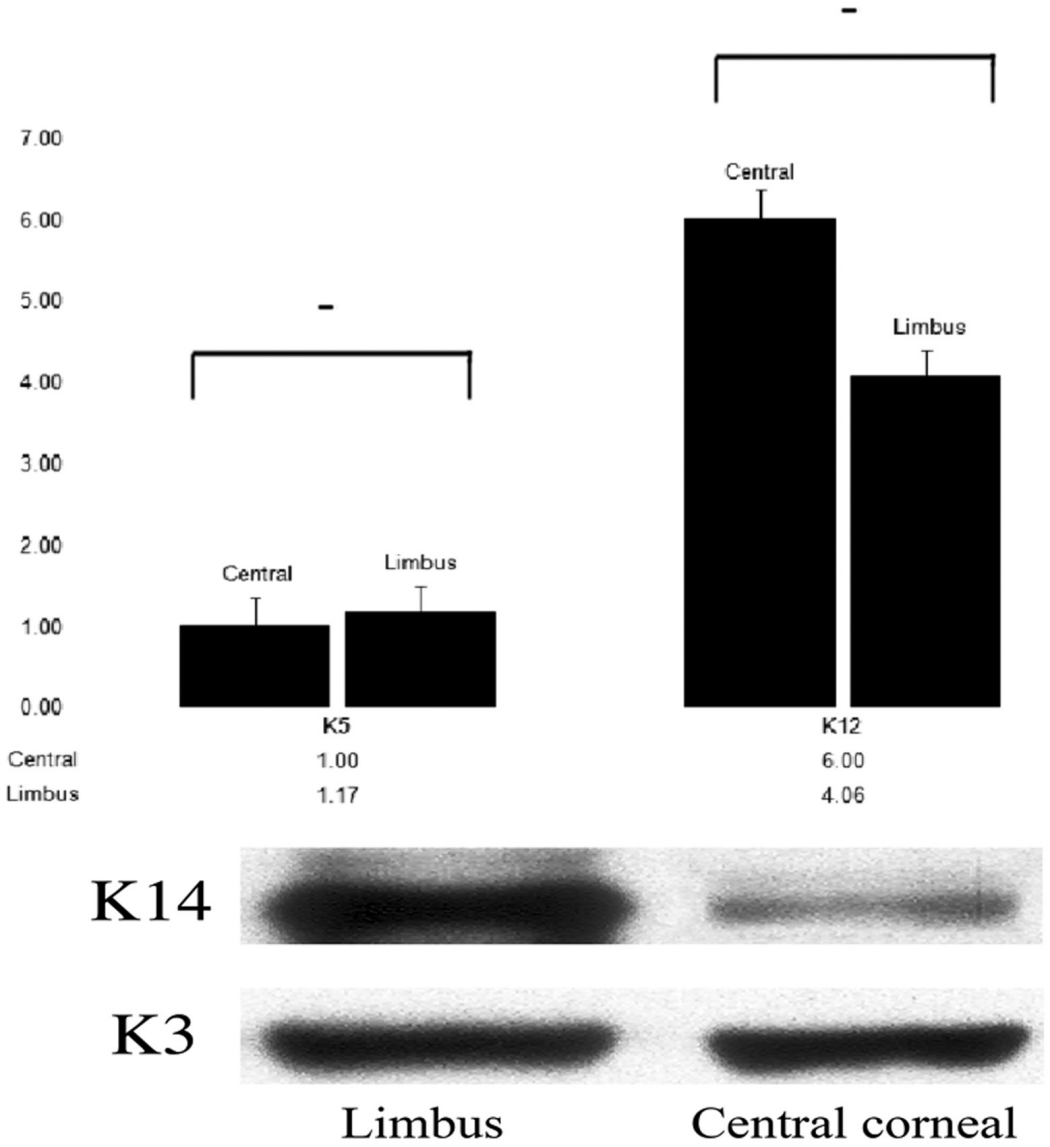
A comparison of K5/14 and K3/12 gene and protein expression in intact bovine limbus and central cornea. Real-time PCR results demonstrated that K5 mRNA level in limbus and central cornea is not statistically significant (A). However, K14 protein in the limbus showed higher expression than the central cornea (B). K12 mRNA (A) and K3 protein (B) showed similar expression level both in central and limbal cornea.

To quantify mRNA levels of K12 (works as a pair with K3) and K5 (works as a pair with K14) expression, we applied real-time PCR. In general, K5 mRNA had a similar expression level in central cornea and limbus (Figure 2); and K12 mRNA expression level was also found not to be significantly different between central cornea and limbus.

Western blotting was used to examine K3 and K14 protein expression. K14 protein level expression was clearly higher in limbus than in central cornea. K3 protein level showed strong expression both in limbus and central cornea (Figure 2).

### K14 expression in cultured epithelial cells before and after air-lifting

**Figure 3:**
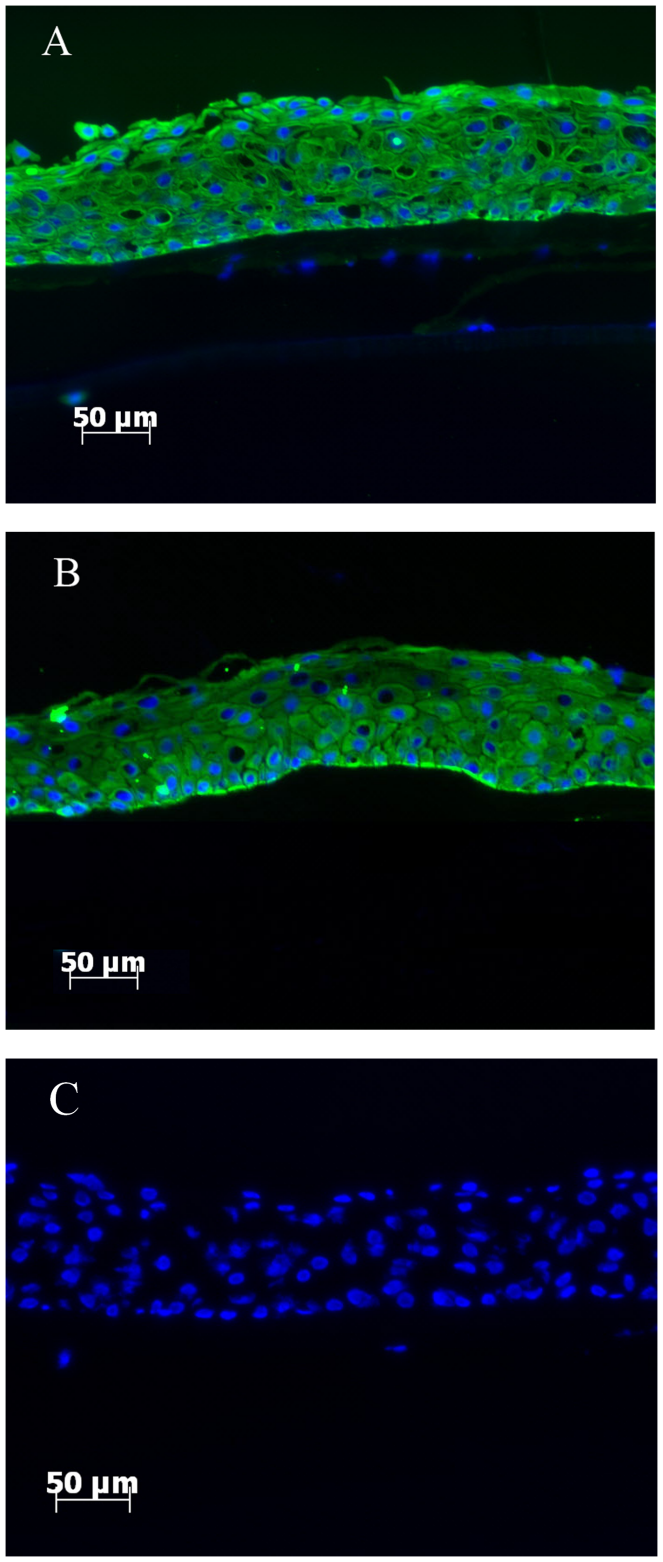
Air-lifting treatment had limited effect on K14 expression in cultured cells. K14 expression in limbal epithelial cells cultured on amniotic membrane before air-lifting (A) and after air-lifting (B). No labeling was seen in the control section (C).

Bovine limbal epithelial cells were isolated and then cultured on intact AM for two weeks plus a further week following air-lifting, resulting in stratification of the cultured cells into 5–7 cell layers. K14 expression was observed throughout all cell layers after 2 weeks in culture (Figure 3A). Following air-lifting, K14 expression appeared slightly reduced in the superior cell layers, but was still strongly expressed in the basal cell layers (Figure 3B).

**Figure 4:**
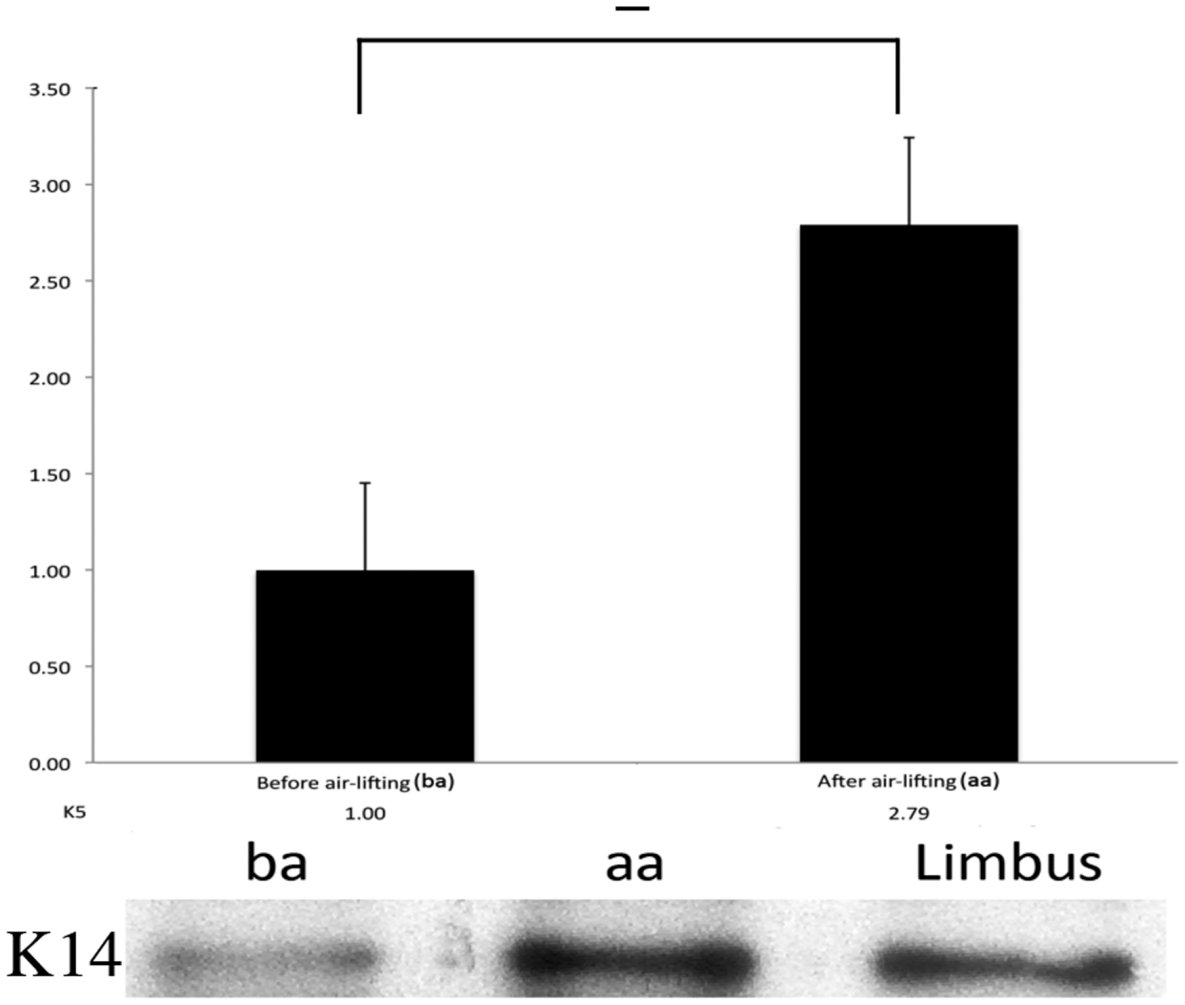
The effect of air-lifting treatment on cells cultured on amniotic membrane. Following air-lifting, K5 mRNA level in the cultured cells increased nearly 3 fold (A), and K14 protein showed a clear increase in its level after air-lifting treatment (B). (ba: before air-lifting, aa: after air-lifting).

Real-time PCR results demonstrated that in cultured limbal epithelial cells, after a week air-lifting, K5 mRNA expression level had slightly increased (but not statistically significant). This observation was also confirmed by K14 protein expression, *i.e.* western blotting results showed that K14 protein expression increased after air-lifting, which mirrored the results obtained from real-time PCR (Figure 4).

## Discussion

Corneal epithelial stem cells have been proposed to be exclusively located to the basal layer of limbal region [22], [32], [33], [34]. K14 expression, as a proposed corneal epithelial stem cell marker, highlights the basal cell layer within the corneal limbus [10], but information on its expression in the central cornea is limited. K3 is a differentiated cell marker [11], which is usually found expressed in superior layers of limbal epithelium, and throughout central corneal epithelium. However our results, using multiple approaches, demonstrate that within intact bovine cornea, K14 expression is not only located to the basal layer of limbal epithelium, but is also found in the central corneal epithelium, similar to K3 expression pattern.

In our studies of K14 protein distribution across the ocular surface, immunohistochemistry (IHC) results clearly showed that K14 positive cells covered the majority of limbal basal layer cells, and also all cell layers in central cornea. These results suggest that either i) corneal epithelial stem cells are not restricted to the limbal basal layers; or ii) K14 is not a precise corneal epithelial stem cell marker. Corneal epithelial stem cells have recently been proposed to be distributed throughout cornea, implying that the corneal limbus is not the only niche for corneal stem cells [4]. Based on this hypothesis, the stem cell markers should be able to detect stem cells not only located in the limbal basal cell layer, but also multiple locations throughout cornea. Our K14 expression fit this hypothesis, *i.e.* expression is not restricted to the limbus region, but is also found to be positive in central cornea. Therefore, this may offer an explanation of our results regarding K14 expression. However, although K14 was located to the basal cell layer of the limbus, it has also been found positive in conjunctiva cells [35], thus, the reliability of applying K14 as a distinct stem cell marker requires further examination.

In our studies we quantified K5/12 mRNA and K3/14 protein level in cornea. Real-time PCR (RTPCR) results demonstrated that the K5/K12 mRNA relative expression ratio is more than 1/6 (∼17%) higher in the limbus, and western blotting (WB) results showed the K14 expression is also higher than K3 in limbus. Although the ratio between stem cells and differentiated cells in corneal limbus is not well defined, it is generally accepted that only 10% of limbal *basal* cells are stem cells [1]. Therefore, K5/14 pair is expressed in more than just corneal epithelial stem cells, and must also include a certain amount of differentiated corneal epithelial cells. K5/14 positive cells are likely to include cells in varying states of differentiation *i.e.* from stem cells to transit amplifying cells (TAC) and finally to terminal differentiated cells. Throughout this process a cell may express several different profiles of differentiation and progenitor markers at any one moment within a tissue.

Air-lifting, as a cell culture technique, was originally developed to create sheets of skin cells for transplantation and to promote epidermal cell differentiation [27], [28], this technique has since been applied successfully to corneal cell culture [18]. To further investigate the K5/14 as a stem cell marker, we cultured limbal epithelial cells on AM for 2 weeks, and then artificially promoted cell differentiation by employing the air-lifting technique. Interestingly, IHC results showed that air-lifting had a limited effect on K14 protein amount and distribution, and RTPCR and WB results showed that both K5 mRNA level and K14 protein level were actually slightly increased after air-lifting. Therefore, these results appear counter intuitive since air-lifting is thought to increase the level of differentiation in these cells. These lines of evidence suggest that the observed multiple locations of K14 positive cells is not because the corneal stem cells reside in more than one niche, in contrast, it is more likely due to the dynamic state of the corneal epithelial stem cells differentiation states.

In conclusion, K14 has been proposed as a limbal stem cell marker for mammalian cornea, and this is consistent with our results, since K14 highlighted the basal layer of the bovine limbal region (an area known to contain epithelial stem cells). Therefore, K14 can be thought of as a stem cell marker under specific circumstances, i.e. within the limbus of a quiescent cornea. However, this paper also used multiple lines of evidence to demonstrate that K14 expression is not always specific to limbal stem cells, as its accuracy is degraded in cells actively undergoing differentiation. In summary, our results highlight the fact that a decisive molecular corneal stem cell marker remains out of reach and the difficulties of identifying robust stem cell markers is hampered by the inherent dynamic status of the stem cell.
